# Power, anxiety, and conflict: how dominative and cooperative orientations shape attitudes toward international relations

**DOI:** 10.3389/fpsyg.2026.1770594

**Published:** 2026-04-20

**Authors:** Yuchen Xiao, Qi Cao, Rongrong Wan, Hui-Ling Hu

**Affiliations:** 1 Law School, Jiulonghu Campus, Southeast University, Nanjing, China; 2 Graduate School of International Studies, Hanyang University, Seoul, Republic of Korea; 3 Middlebury Institute of International Studies at Monterey, Middlebury College, Monterey, CA, United States; 4 Institute of Creative Design and Management, National Taipei University of Business, Taoyuan, Taiwan, China

**Keywords:** attitudes toward international conflict, cooperative power orientation, dominative power orientation, national security anxiety, political psychology

## Abstract

**Introduction:**

This study examines the associations between dominative and cooperative power orientations and individuals’ attitudes toward international conflict, with national security anxiety serving as a mediating variable.

**Methods:**

Based on a survey of 393 Chinese university students, structural equation modeling (SEM) was employed to test the proposed framework.

**Results:**

The results show that (1) dominative power orientation positively predicts national security anxiety, which in turn increases support for militarized responses; (2) cooperative power orientation reduces security anxiety and enhances preference for diplomatic and peaceful approaches; and (3) national security anxiety plays a significant mediating role between power orientation and conflict attitudes.

**Discussion:**

This research bridges international relations theory and political psychology by revealing how power beliefs shape emotional and attitudinal processes at the individual level. The findings also provide practical implications for national security education and public communication, encouraging a more balanced and cooperative understanding of power and security among youth. This study examines the associations between dominative and cooperative power orientations and individuals’ attitudes toward international conflict, with national security anxiety serving as a mediating variable. Based on a survey of 393 Chinese university students, structural equation modeling (SEM) was employed to test the proposed framework. The results show that (1) a dominative power orientation positively predicts national security anxiety, which in turn increases support for militarized responses; (2) a cooperative power orientation reduces security anxiety and enhances preference for diplomatic and peaceful approaches; and (3) national security anxiety plays a significant mediating role between power orientation and conflict attitudes. Theoretically, this research bridges international relations theory and political psychology by revealing how power beliefs shape emotional and attitudinal processes at the individual level. Practically, the findings provide valuable implications for national security education and public communication, encouraging a more balanced and cooperative understanding of power and security among youth.

## 1 Introduction

In the historical development of international relations theory, power has consistently remained a core concept for explaining international political phenomena. From realism, which emphasizes states’ power-oriented and self-interested behavior, to neoliberal institutionalism, which focuses on how institutional power constrains anarchy, different interpretations of power have shaped major paradigmatic shifts within the discipline (Baldwin, 2016). Traditional realism regards power as a resource of control and domination, particularly emphasizing the importance of military and economic capability. It assumes that states must enhance their relative advantages to survive and secure sovereignty (Mearsheimer, 2019). Morgenthau and Nations (1948) defined power as “control over the actions of others,” while Wallace (1971) argued that the anarchic structure of the international system compels states to pursue relative power positions to cope with external uncertainty. This perspective—centered on *dominative power* (power over)—has long underpinned realism’s explanatory logic of international behavior and continues to influence post–Cold War security policies. Contemporary examples such as the Russia–Ukraine war and the U.S.–China strategic rivalry illustrate that armament and deterrence remain central to most national strategies (Schweller, 2016). However, in the 21st century, as the international community faces increasing multipolarity, non-traditional security threats, and frequent transnational crises, the singular notion of “power as control” has become insufficient to explain the diverse and complex interactions among states. Contemporary scholars such as Keivan Hosseiny (2021) and Adler-Nissen and Pouliot (2014) argue that power should instead be understood as *relational practice*, emphasizing that power is not a quantifiable aggregate of resources but a social process constructed through language, norms, and identity. This perspective moves beyond the materialist conception of power, highlighting its interactive and socially constructed nature.

Building upon this foundation, feminist and constructivist theories further critique and reconstruct mainstream conceptions of power. Feminist international relations theory argues that traditional understandings of power have long been dominated by a male-centered perspective, which confines power to domination and coercion while neglecting alternative forms such as cooperation, empowerment, and reciprocity. Tickner and True (2018) contend that power should be redefined as “the ability to empower others and act together” (*power to* and *power with*), rather than merely “the ability to impose one’s will on others.” Constructivism further extends this critique by emphasizing that states’ threat perceptions and behavioral preferences are not determined solely by structural constraints but are socially shaped through identity, historical experience, and interaction (Senlin, 2019). In the context of emerging global challenges such as the COVID-19 pandemic, climate change, and human security, the cooperative conception of power provides a more appropriate explanatory lens. It highlights the importance of understanding international relations not only through material capabilities but also through shared norms and interdependence. This shift underscores the need for international relations scholarship to incorporate nontraditional, relational forms of power more comprehensively (Billiet et al., 2021; Lin et al., 2021).

Nevertheless, existing literature remains largely confined to theoretical dialogue and critique, with insufficient attention to the cognitive and psychological mechanisms of power orientation at the individual level. Although traditional realism and feminist theory hold divergent views on the nature of power, most studies continue to focus on macro-level structures and state-level analysis, rarely exploring how power orientations translate into individuals’ specific attitudes toward international politics. For instance, there is a lack of empirical and quantitative research examining how dominative and cooperative power orientations influence personal views on war and diplomacy. Moreover, recent studies in political psychology indicate that national security anxiet*y* plays a crucial mediating role in shaping collective support for military action and attitudes toward international conflict (Gillath and Hart, 2010; Gross et al., 2017; O’Brien and Tropp, 2015). However, whether power orientations affect conflict attitudes through the mediation of anxiety has not yet been systematically tested. In other words, the current academic field lacks an integrated structural model linking power orientation → emotional cognition → conflict attitudes, and few studies have compared the psychological mechanisms underlying dominative versus cooperative power orientations. This gap between theoretical discourse and empirical validation underscores the need for a comprehensive framework that bridges power theory and political psychology at the individual level.

Building upon the aforementioned theoretical development and research gaps, this study aims to construct a mediational model in which dominative and cooperative power orientations serve as antecedent variables, national security anxiety functions as the mediating variable, and attitudes toward international conflict represent the outcome variable. Through quantitative survey data and structural equation modeling (SEM) analysis, this research seeks to examine how different types of power orientations are associated with individuals’ attitudes toward international conflict through emotional responses. Given the study’s focus on individual-level cognition and emotional mechanisms, university students are selected as the target population. As a key group in the early stage of political socialization, they possess both high cognitive plasticity and theoretical representativeness. According to political socialization theory, the youth period is a critical stage for the formation of power beliefs and international attitudes, which exert lasting influences on future political behaviors and policy preferences. Moreover, university students are cognitively capable of comprehending abstract concepts such as dominative power, cooperative power, and national security anxiety, thus providing reliable responses to quantitative measures of these constructs—a practice widely adopted in related empirical studies. By employing this research design, the study aims to fill the gap in traditional international relations theories regarding micro-level psychological mechanisms, offering an interdisciplinary framework that integrates insights from international relations, political psychology, and peace studies. Unlike prior international relations research, which has been largely qualitative and theory-driven, this study introduces a quantitative mediational model to empirically examine the psychological mechanisms linking power orientations to conflict attitudes at the individual level. Given the current geopolitical tensions and the increasing prominence of major power competition, particularly involving China, the issue of national security anxiety has become especially salient among younger generations. Understanding how such anxiety shapes attitudes toward international conflict is therefore both theoretically and practically important.

## 2 Literature review

### 2.1 The evolution of power conceptions in international relations theory

#### 2.1.1 Classical realism and the concept of dominative power (*power over*)

In the early development of international relations theory, realism occupied a central position, and its interpretation of “power” profoundly shaped post–Cold War international political thinking and national security policies. Classical realists such as Morgenthau and Nations (1948) argued that the essence of international politics lies in power politics, where state actors—operating within an anarchic international system—must inevitably seek control over the behavior of others in order to survive. Morgenthau defined power as “the ability to influence or control the actions of others,” a notion that forms the foundation of the concept of dominative *power* (power over). The core logic of this view posits that only through domination and control can states safeguard their interests and ensure their own security.

Building further on this perspective, Waltz (1979), in his structural realism, argued that the anarchic nature of the international system and the unequal distribution of power compel states to pursue relative power in order to maintain their status and prevent external aggression. He emphasized that the driving forces of international relations lie not in intentions or morality but in structural constraints that foster “self-help behavior” and the balance of power. This view—measuring power primarily in terms of military and economic capabilities—highlights zero-sum competition and power balancing as defining features of dominative power at the state level. The concept of dominative power has profoundly influenced security strategies and military thinking since the Cold War. For example, Mearsheimer (2019), in offensive realism, contends that states, in their quest for security, tend to expand their power continuously to deter the rise of potential adversaries, resulting in an enduring pattern of arms races and deterrence policies. Similarly, Schweller (2016) notes that great powers maintain their dominance and geopolitical advantages through military deployments and strategic alliances—practical manifestations of dominative power in contemporary international politics.

In summary, classical realism conceptualizes power as a measurable and comparable material resource—such as military and economic capabilities—and emphasizes the control and suppression of opponents as the means to safeguard national interests. This conception of power, centered on “power over,” not only dominated international relations theory during the Cold War era but also continues to shape mainstream understandings of security policy and international conflict today.

#### 2.1.2 Neoliberal institutionalism and institutional power

In contrast to classical realism—which views power as a zero-sum competition centered on control over resources and the pursuit of dominance—neoliberal institutionalism emphasizes that, even within an anarchic international system, states can cooperate through international institutions and rules to reduce conflict and expand mutual gains. This perspective, established by Keohane and Nye (1973), argues that institutions can promote transparency, lower transaction costs, and foster mechanisms of trust even in the absence of a central authority, thereby reshaping state actors’ preferences and strategies (Keohane, 2005).

From the perspective of neoliberal institutionalism, power is manifested not only through military and economic strength but also through international institutions and norms—commonly referred to as institutional power. Keohane (1984) argued that power can be exercised through institutional design; for instance, agenda setting, rule making, and institutional exclusivity can all serve to reinforce the advantages of certain states. This form of power differs from the direct control associated with dominative power, representing instead an indirect form of influence embedded within institutional structures. Institutional power also involves the asymmetrical interactions between “rule-makers” and “rule-takers.” Barnett and Duvall (2005) categorize this as institutional power, emphasizing that institutions are not neutral intermediaries but rather contain inherent power configurations that can reshape the distribution of power and the modes of interaction among states. This perspective underscores that power is not merely the outcome of resource possession but also a long-term effect produced by asymmetrical rules operating within institutional frameworks.

In contemporary international politics, institutional power has become widely evident in international organizations, trade agreements, and multilateral mechanisms. For example, the institutional influence of the United States and the European Union within organizations such as the World Trade Organization (WTO) and the International Monetary Fund (IMF) often allows them to shape the content of rules and the distribution of resources, thereby exerting both normative constraints and structural effects on other states’ behavior. In summary, neoliberal institutionalism broadens the understanding of power beyond material control and coercion, focusing instead on how institutions function as vehicles of influence in shaping international interactions and national choices. This perspective provides a crucial theoretical foundation for constructivist interpretations of the relational and social dimensions of power that emerged in subsequent developments within international relations theory.

#### 2.1.3 Constructivism and the relational conception of power (*power as practice*)

Constructivism, which emerged in the 1990s, marked the “social turn” in international relations theory and challenged the traditional perspectives of realism and neoliberal institutionalism that regarded power as either a material resource or an instrument of institutional design. Constructivist scholars such as Wendt (1992) argue that international politics is not determined unilaterally by objective structures or material forces but is co-constructed by actors through ideas, norms, and identities in their interactions. In other words, power is no longer merely a means of control or coercion but a process that is continuously reproduced and imbued with meaning through social practice (Adler and Pouliot, 2011).

Within this framework, relational power *or* power as practice has become a central constructivist interpretation of the concept of power. Adler and Pouliot (2011) argue that power should be understood as a social practice, manifested in the ongoing processes of interaction between actors. This perspective transforms power from a static possession into a dynamic form of action, emphasizing its generative and co-constitutive nature. When states or individuals display specific postures, gestures, or linguistic expressions in diplomacy, negotiation, or even everyday communication, they are, in effect, enacting power. Hence, power is not confined to material superiority but is embedded in the reproduction of social relations, symbolic meanings, and patterns of interaction.

The relational conception of power begins with the premise that power exists within relationships (power-in-relations) rather than residing within individual actors. As Keivan (2021) explains, power is a bidirectional and interactive process that can be exercised not only through acts of domination but also through recognition, norms, and cooperation. This implies that the stability and conflict of international politics are determined not merely by military or economic resources, but by how actors interpret each other’s intentions and role positions within social interactions. For instance, Adler-Nissen and Pouliot’s (2014) analysis of the negotiations surrounding the international intervention in Libya demonstrates that diplomats’ identity recognition, discursive authority, and symbolic performances often provide better explanations of negotiation outcomes than formal institutional arrangements or military capabilities.

Moreover, constructivism emphasizes the constitutive role of language and symbols in shaping power relations. Barnett and Duvall’s (2005) concept of productive power highlights that power operates not merely through command and obedience but through discourse that defines what is considered possible, legitimate, and imaginable. In this sense, power becomes the capacity to shape reality rather than merely a tool of control. When the international community frames norms and behaviors through specific discursive constructs—such as “humanitarian intervention,” “global governance,” or the “community of shared destiny”—it is exercising a form of relational power whose effects are far-reaching and socially reproductive.

In sum, constructivism’s relational conception of power views power as an ongoing interactive practice shaped by social structures, identities, and discursive systems. This perspective transcends the traditional materialist framework that equates power with resources, offering a new lens for understanding how international actors construct power orders through everyday interactions. For the present study, the constructivist relational approach reveals the social foundations of cooperative power and provides a theoretical basis for exploring how individual-level power orientations influence attitudes toward international conflict.

#### 2.1.4 Feminist reinterpretations of power (power to/power with)

Since the 1980s, feminist international relations theory has offered a profound critique of traditional conceptions of power, arguing that mainstream IR theories have long been dominated by androcentrism—a male-centered worldview that overemphasizes the dominative (power over), competitive, and violent dimensions of power while neglecting its cooperative, caring, and empowering potentials (Enloe, 2014; Tickner, 1992). Feminist scholars contend that realism’s conflation of “power” with “domination” not only reproduces gendered hierarchies of power but also obscures the constructive functions of power in fostering collective action and social connectedness (Peterson, 2004).

Theoretically, feminist scholars redefine the nature of power by distinguishing between “power to” and “power with.” Young (1990) explains that power to emphasizes the capacity or potential of individuals or groups to realize their goals and act upon them—it represents a creative and generative form of power. In contrast, power with focuses on the reciprocal strength that emerges from collaboration and collective action, symbolizing a relational and interdependent understanding of power. Compared with the coercive logic of power over, these perspectives conceptualize power as a process that promotes social transformation and collective empowerment, reflecting the feminist political ethic that power can be shared (Allen, 2016). Tickner (1992), in Gender in International Relations, further argues that traditional international politics has long associated “security” and “strength” with masculinized militarism, thereby neglecting cooperation and care as foundational elements in the construction of security. She advocates replacing “domination and deterrence” with “empowerment and collective agency” as the core of understanding power, thereby challenging the state-centered security logic grounded in friend–enemy dichotomies. This perspective not only exposes the gendered construction of power but also provides the theoretical foundation for the notion of cooperative power, emphasizing non-dominative influence formed through trust, consensus, and emotional connection.

Feminist reconceptualizations of power also emphasize the roles of language and emotion in power interactions. As Sylvester (1994) argues, the relational practices of international politics are not merely the outcomes of institutional arrangements and material interests but are also social processes shaped by emotion, care, and mutual understanding. This view resonates with the constructivist notion of power as practice: both perspectives focus on how power is generated through interaction and jointly reject static or materialist interpretations of power. The distinction, however, lies in feminism’s integration of ethics and the ethic of care into power theory, underscoring that power should serve the advancement of human well-being and social justice.

In sum, the feminist reconstruction of power fundamentally challenges the traditional logic of “power as control” and proposes an alternative understanding centered on empowerment and cooperation. This perspective not only broadens the conceptual boundaries of power but also lays the theoretical and normative foundation for the subsequent development of the concept of cooperative power. For the present study, the feminist view of power suggests that power should not be regarded as a zero-sum contest but as a relational resource that fosters collective security, social trust, and peaceful coexistence.

### 2.2 Theoretical distinctions and operationalization of dominative and cooperative power orientations

Building upon the preceding theoretical traditions, Dominative Power Orientation and Cooperative Power Orientation can be conceptualized as two psycho-cultural archetypes of the broader notion of power. They reflect fundamentally different belief systems concerning whether power exists to control others or to act together with others. The former, rooted in realism and traditional security paradigms, emphasizes competition, deterrence, and the pursuit of superiority. The latter, inspired by constructivist and feminist thought, posits that power can be realized through trust, collaboration, and the achievement of shared goals. These two orientations differ not only at the theoretical level but may also influence individuals’ political attitudes, foreign policy preferences, and conflict response patterns.

#### 2.2.1 Cognitive foundations and behavioral tendencies of dominative power orientation

The Dominative Power Orientation originates from the realist assumption regarding the nature of international politics—namely, that the international system is characterized by anarchy, where states compete with one another in an environment of mutual distrust. Under such conditions, power becomes the primary instrument for ensuring survival and maintaining security (Morgenthau et al., 1985; Quinn and Gibson, 2017). At the psychological level, this orientation corresponds to the constructs of Social Dominance Orientation (SDO) and Authoritarianism (Altemeyer, 1996; Pratto et al., 1994). Individuals high in dominative power orientation tend to perceive both social and international interactions as hierarchical structures in which the strong must exercise control over others to preserve order and stability.

Cognitively, the dominative power orientation is grounded in zero-sum thinking—the belief that “one party’s gain necessarily entails another’s loss.” Such a mindset leads individuals to focus on threat perception and defensive attributions when interpreting international issues, thereby reinforcing vigilance and hostility toward external groups (Duckitt, 2001). Behaviorally, individuals high in dominative power orientation are more inclined to support military intervention, assertive diplomacy, and unilateral policies, viewing power as the principal means to secure national interests and maintain sovereignty (Mearsheimer, 2019). This orientation aligns with the logic of offensive realism, which posits that proactive expansion and deterrence are essential strategies for preventing potential threats.

Furthermore, the dominative power orientation is often accompanied by a threat amplification effect. Studies have shown that when individuals are confronted with international crises or security challenges, those with higher dominative tendencies are more prone to experience national security anxiety, which in turn strengthens their support for coercive and hardline policies (Feldman and Stenner, 1997; Huddy et al., 2005). Thus, dominative power orientation represents not only a political attitude but also an emotional-cognitive disposition that integrates psychological needs for authority, order, and strength.

In summary, the core logic of the dominative power orientation consists of three key propositions:

1. The essence of power lies in control and deterrence—ensuring survival and security;
2. The world is perceived as a competitive and hierarchical system—trust is difficult to establish;
3. Behavioral strategies are oriented toward defense and coercion—prioritizing military and unilateral measures to safeguard sovereignty.

At the international level, this orientation corresponds to the realist logic of power politics, whereas at the psychological level, it manifests as a dominance- and threat-oriented mindset. Together, these tendencies exert a strong positive influence on individuals’ attitudes toward international conflict.

#### 2.2.2 Social construction and practical characteristics of cooperative power orientation

In contrast to the dominative power orientation—which views power as a tool for controlling others to ensure survival—the Cooperative Power Orientation emphasizes the capacity to “achieve shared goals through relationships.” Its theoretical roots can be traced to constructivist and feminist perspectives in international relations (Adler and Pouliot, 2011; Tickner, 1992). From this standpoint, power is no longer understood as a scarce resource but rather as a shared social practice, manifested through processes of mutual trust, collaboration, and consensus-building (Barnett and Duvall, 2005).

Constructivist scholars argue that the operation of power depends not only on material capabilities but also on how interaction structures and identity constructions among actors are socially constituted (Wendt, 1992). Accordingly, the core assumption of cooperative power orientation is that actors can jointly shape consensus and achieve stability and trust through norms, language, and symbolic actions (Adler-Nissen and Pouliot, 2014). For example, the functioning of international cooperative organizations, transnational climate governance, and global public health coordination mechanisms demonstrates that power can manifest in forms of reciprocity and co-governance rather than domination and subordination.

From a psychological perspective, cooperative power orientation reflects a trust-based worldview that prioritizes interdependence and shared responsibility. Individuals who endorse this orientation tend to interpret international relations through the lens of reciprocity and collective security rather than rivalry or threat. This mindset reduces anxiety and defensive cognition, fostering openness to dialogue and compromise (Deutsch, 2011). Empirical studies on cooperative problem-solving and peace psychology suggest that cooperation-oriented individuals are more likely to support multilateralism, humanitarian engagement, and confidence-building measures (Kelman, 2010; Nye, 2011). In practice, cooperative power manifests as “soft power” or “relational influence”—the ability to attract, persuade, and inspire others through legitimacy and shared values rather than coercion (Nye, 2011).

In essence, cooperative power orientation represents a non-zero-sum understanding of power. It emphasizes that influence arises from the capacity to generate mutual benefits and shared meaning within social interactions. By situating power within communicative and ethical relationships, this perspective aligns with feminist notions of power with and constructivist views of power as practice. Together, they redefine power as a generative and relational process that sustains peace, trust, and long-term international stability.

Empirical evidence also supports the effects of cooperative power orientation. Individuals with a stronger cooperative orientation tend to exhibit greater altruistic behavior, a preference for collective decision-making, and higher levels of trust and reciprocity in social interactions (Balliet et al., 2011; Eagly and Wood, 1991). Research in social and political psychology further indicates that cooperative orientations are associated with support for peaceful conflict resolution and reduced intergroup hostility (De Dreu, 2010; Roccas et al., 2006). In international contexts, cooperative beliefs about power have been shown to promote willingness for transnational collaboration and multilateral engagement, particularly when addressing global challenges such as climate change, pandemic governance, and peacebuilding, while simultaneously reducing international anxiety and hostility (Messner et al., 2015; Romano et al., 2017). These findings suggest that cooperative power orientation is not merely a theoretical construct but also reflects an increasingly important logic of governance in contemporary international society.

In summary, the cooperative power orientation is characterized by three key features:

1. Relational and generative nature of power—power exists within interaction and collaboration;
2. Shared and reciprocal quality of power—emphasizing mutual interests rather than zero-sum competition;
3. Ethical orientation of power—centering on empowerment, care, and peace as core values.

This perspective not only redefines the essence of power but also provides a new psychosocial foundation for understanding individuals’ attitudes and behaviors in international affairs—particularly how cooperative beliefs can alleviate security anxiety and foster peaceful orientations.

### 2.3 The psychological foundations of national security anxiety

Within the framework of political psychology, anxiety is understood as an emotional response to uncertainty and perceived threat, serving the adaptive function of enhancing vigilance and facilitating defensive decision-making (Marcus et al., 2000). When individuals confront potential political or security threats, anxiety activates a surveillance system that heightens attention to risks, motivates information seeking, and increases support for assertive or protective policies (Huddy et al., 2005). This emotional process can be categorized into two types: the first, specific anxiety, refers to reactions toward identifiable events or hostile actions; the second, diffuse anxiety, arises from a prolonged sense of insecurity and perceptions of social instability (Albertson and Gadarian, 2015).

The concept of national security anxiety (NSA) corresponds to the latter form of anxiety, referring to individuals’ persistent sense of unease and apprehension regarding national survival, sovereignty, and external threats. Research suggests that the formation of security anxiety involves three key mechanisms. The first is threat framing in media messages and political discourse, which amplifies public perceptions of a hostile environment. The second involves psychological projection of identity and group boundaries, linking national security to personal security at the individual level. The third is the mediating role of power orientation—individuals with a high dominative power orientation, emphasizing control and deterrence, tend to experience stronger anxiety in response to potential threats (Gillath and Hart, 2010; Gross et al., 2017).

National security anxiety refers to individuals’ emotional responses to perceived threats in the international environment, reflecting both cognitive evaluations of risk and affective reactions to uncertainty. A growing body of research highlights the mediating role of affect in shaping political and international attitudes. Emotions such as anxiety, fear, and anger serve as key mechanisms through which cognitive beliefs are translated into policy preferences (Brader, 2020; Marcus et al., 2000). In the context of international conflict, anxiety has been shown to increase threat sensitivity and support for defensive or aggressive policies, while also influencing information processing and risk perception (Albertson and Gadarian, 2015; Gross et al., 2011). Building on this perspective, this study conceptualizes national security anxiety as a key psychological mechanism linking power orientations to attitudes toward international conflict.

In terms of measurement, national security anxiety is commonly assessed using self-report scales that include items such as concerns about international instability, worries about national defense preparedness, and fears of sovereignty infringement (Huddy et al., 2005). These emotional indicators not only reflect individuals’ psychological sensitivity to external threats but also predict their attitudinal tendencies regarding foreign and military issues. Empirical studies have shown that security anxiety is positively correlated with support for war and distrust toward hostile groups, while negatively associated with diplomatic negotiation and willingness for international cooperation (Brader et al., 2008). In other words, anxiety functions as a mobilizing emotion in political decision-making, influencing policy preferences and attitudes toward international conflict through perceived threat.

Overall, national security anxiety embodies both cognitive and emotional dimensions, serving as a crucial psychological mechanism linking individual power orientations to conflict attitudes. It not only reflects people’s threat evaluations of the external environment but also reveals how emotion operates as a cognitive frame mediating between dominative and cooperative power orientations. This perspective contributes to security studies by offering a micro-level psychological foundation for understanding national security attitudes.

### 2.4 Factors influencing attitudes toward international conflict

Attitudes toward international conflict refer to individuals’ evaluative and affective orientations toward international behavioral options such as military action, diplomatic negotiation, and peace mechanisms (Myton, 2009). Research in political psychology and international relations generally contends that the formation of conflict attitudes arises from the interplay of multiple psychological, social, and cognitive factors. A substantial body of research in political psychology has further examined the distinction between internationalism and militarism in shaping foreign policy attitudes. Individuals with militaristic or hawkish orientations tend to support the use of force and prioritize national strength, whereas those with internationalist orientations are more likely to favor diplomacy, cooperation, and multilateral institutions (Herrmann et al., 1999; Wittkopf, 1990). These attitudinal differences reflect underlying belief systems about the role of power and the legitimacy of military action in international affairs.

At the cognitive level, individuals’ worldviews and power orientations serve as critical frameworks for interpreting international disputes. Those with a high dominative power orientation tend to perceive international relations as a zero-sum game characterized by competition and threat, leading to stronger support for assertive diplomacy and military action. Conversely, individuals with a high cooperative power orientation are more inclined to favor multilateral negotiations, diplomatic dialogue, and institutionalized cooperation as means of conflict resolution (Mearsheimer, 2019; Tickner, 1992).

At the emotional level, affect plays a crucial mediating role in shaping conflict attitudes. Studies have shown that national security anxiety, fear, and perceived threat heighten support for defensive policies and military mobilization while reducing trust in diplomatic compromise (Gross et al., 2017; Huddy et al., 2005). In contrast, positive emotions such as trust and empathy help mitigate hostile biases and enhance individuals’ endorsement of international cooperation and peace mechanisms (Halperin, 2011).

At the social level, group identity and media narratives also play a crucial role in shaping public attitudes toward international conflict. Social identity theory suggests that individuals derive part of their self-concept from group membership, which can lead to in-group favoritism and out-group bias (Tajfel et al., 2001). Empirical studies have shown that stronger national identification is associated with higher levels of perceived threat, greater support for military action, and more adversarial attitudes toward out-groups (Huddy, 2001; Roccas et al., 2006). Stronger national identification tends to evoke defensive nationalism, prompting individuals to adopt more adversarial stances during international disputes. Meanwhile, threat framing and war metaphors in media coverage can reinforce perceptions of external enemies, thereby legitimizing the use of military action (Nabi, 2003). In addition, social variables such as educational attainment, political efficacy, and international experience have been empirically linked to variations in conflict attitudes (McDermott, 2011).

Overall, the formation of attitudes toward international conflict can be understood as the outcome of three interrelated layers—power orientation, emotion, and social identity. Individuals with a dominative power orientation and higher levels of security anxiety tend to support the use of military force, whereas those with a cooperative power orientation and lower anxiety levels prefer institutional solutions and diplomatic negotiations. This integrative theoretical framework provides a vital foundation for examining the individual-level pathways linking power orientation, national security anxiety, and attitudes toward international conflict.

### 2.5 The integrative framework of power orientation, emotion, and conflict attitudes

#### 2.5.1 Theoretical logic of the model construction

This study constructs an integrative analytical framework of “Power Orientation–Emotion–Conflict Attitudes” to examine how individuals form attitudes toward international conflict through cognitive beliefs and emotional processes. The overall logic is grounded in the “Cognition–Emotion–Behavior” psychological process model (Marcus et al., 2000), in which power orientation can be conceptualized as an upstream cognitive structure, national security anxiety as an intermediary emotional response, and attitudes toward international conflict as the outcome.

At the theoretical level, the Dominative Power Orientation (DPO) reflects the central tenets of realism, which assumes that the international system is an anarchic and competitive arena where states must pursue control over others to ensure survival (Mearsheimer, 2019; Waltz, 1979). Such a worldview tends to trigger heightened threat perception and defensive anxiety. In contrast, the Cooperative Power Orientation (CPO) is rooted in constructivist and feminist relational interpretations of power, which posit that power can be realized through norms, mutual trust, and collective action to achieve shared security (Adler and Pouliot, 2011; Tickner, 1992). This orientation is therefore associated with reduced anxiety and diminished hostility.

At the emotional level, political psychology identifies anxiety as a key mobilizing emotion that emerges from threat evaluation and motivates defensive behavior (Huddy et al., 2005). National Security Anxiety (NSA)—as an emotional response to external uncertainty—drives individuals to seek safety and favor defensive or coercive policies. Individuals experiencing higher anxiety are more likely to support military action and deterrence strategies, whereas those with lower anxiety tend to favor diplomatic negotiation and institutional cooperation (Albertson and Gadarian, 2015). Consequently, anxiety serves as the emotional bridge through which power orientation is transformed into specific conflict attitudes.

The present model assumes that the Dominative Power Orientation (DPO) enhances perceptions of threat and thereby heightens national security anxiety, which in turn promotes hawkish conflict attitudes. In contrast, the Cooperative Power Orientation (CPO) alleviates anxiety by reducing perceived threats and strengthening interpersonal and intergroup trust, thus fostering peace-oriented attitudes. This theoretical chain provides a framework for understanding the potential psychological process of “cognitive belief → emotional response → policy attitude,” thereby addressing the micro-level psychological foundations that traditional international relations theories have largely overlooked.

Moreover, selecting Chinese university students as the research population carries both theoretical and practical significance. From the perspective of political socialization theory (Easton et al., 1969), youth represents a critical period during which political beliefs and international attitudes are formed. As a group characterized by higher education, frequent exposure to information, and high cognitive flexibility, university students can accurately reflect the formative processes of power orientation and security perception. Practically, China’s current rise in global influence amid a highly uncertain international environment makes the younger generation’s security anxiety and international attitudes particularly revealing of the dynamic interplay among national narratives, media framing, and international mindsets. Thus, focusing on university students not only validates the micro-level psychological mechanisms proposed in this study but also provides insight into the security psychology and international behavioral tendencies of China’s new generation within the context of global political transformation.

#### 2.5.2 Research hypotheses on the relationships among variables

Individuals with a Dominative Power Orientation (DPO) tend to perceive the international system as a competitive and antagonistic zero-sum structure (Waltz, 1979), believing that the essence of power relations lies in control and defense (Morgenthau et al., 1985). Such a belief reinforces external threat perception and triggers a hostile attribution bias, making individuals more prone to experience national security anxiety when confronted with international events (Huddy et al., 2005). Furthermore, the defensive logic of realism encourages those with a strong dominative orientation to overestimate potential risks, resulting in a sustained sense of insecurity and anxiety (Gillath and Hart, 2010).

*H1:* Dominative power orientation positively influences national security anxiety.

Individuals with a Cooperative Power Orientation (CPO) emphasize mutual trust and co-governance, preferring dialogue and institutional mechanisms to resolve disputes (Adler-Nissen and Pouliot, 2014). This relational perspective reduces perceptions of external threat while strengthening international trust and a sense of security (Deutsch, 2011). Empirical studies indicate that individuals with a strong cooperative belief system exhibit more stable emotional responses and significantly lower anxiety levels than those with a dominative orientation when facing international uncertainty (Billiet et al., 2021). Therefore, cooperative power orientation can be regarded as an emotional buffer that mitigates national security anxiety.

*H2:* Cooperative power orientation negatively influences national security anxiety.

Research in political psychology suggests that anxiety promotes a defensive decision-making mode, leading individuals to show stronger support for deterrence, military action, and hardline policies (Gross et al., 2017; Marcus et al., 2000). When national security anxiety increases, people tend to rely on force and power balancing as means of reducing uncertainty. Conversely, lower levels of anxiety facilitate rational evaluation and diplomatic trust, thereby decreasing support for military action (Halperin, 2011).

*H3:* National security anxiety positively influences attitudes toward international conflict.

The Dominative Power Orientation (DPO) is rooted in the offensive logic of realism (Mearsheimer, 2019), which posits that security can only be achieved through relative advantage and assertive action. Psychologically, individuals with a dominative orientation tend to regard the use of military force as a legitimate and necessary means to safeguard national status and security. Previous studies have also shown that individuals with high dominance tendencies generally exhibit stronger support for militarism and more hawkish attitudes (Duckitt, 2001; Pratto et al., 1994). Accordingly, it is expected that dominative power orientation will increase support for military action, both directly and indirectly through heightened national security anxiety.

*H4:* National security anxiety mediates the relationship between dominative power orientation and attitudes toward international conflict.

The Cooperative Power Orientation (CPO) reduces threat perception and alleviates anxiety, thereby diminishing support for military action. This mechanism aligns with the emotion-buffer model, which posits that positive beliefs can stabilize emotional responses and, in turn, reduce hostile attitudes (Halperin, 2011). Accordingly, national security anxiety is expected to exert a negative mediating effect in the pathway from CPO to attitudes toward international conflict.

*H5:* National security anxiety mediates the relationship between cooperative power orientation and attitudes toward international conflict.

**Figure 1:**
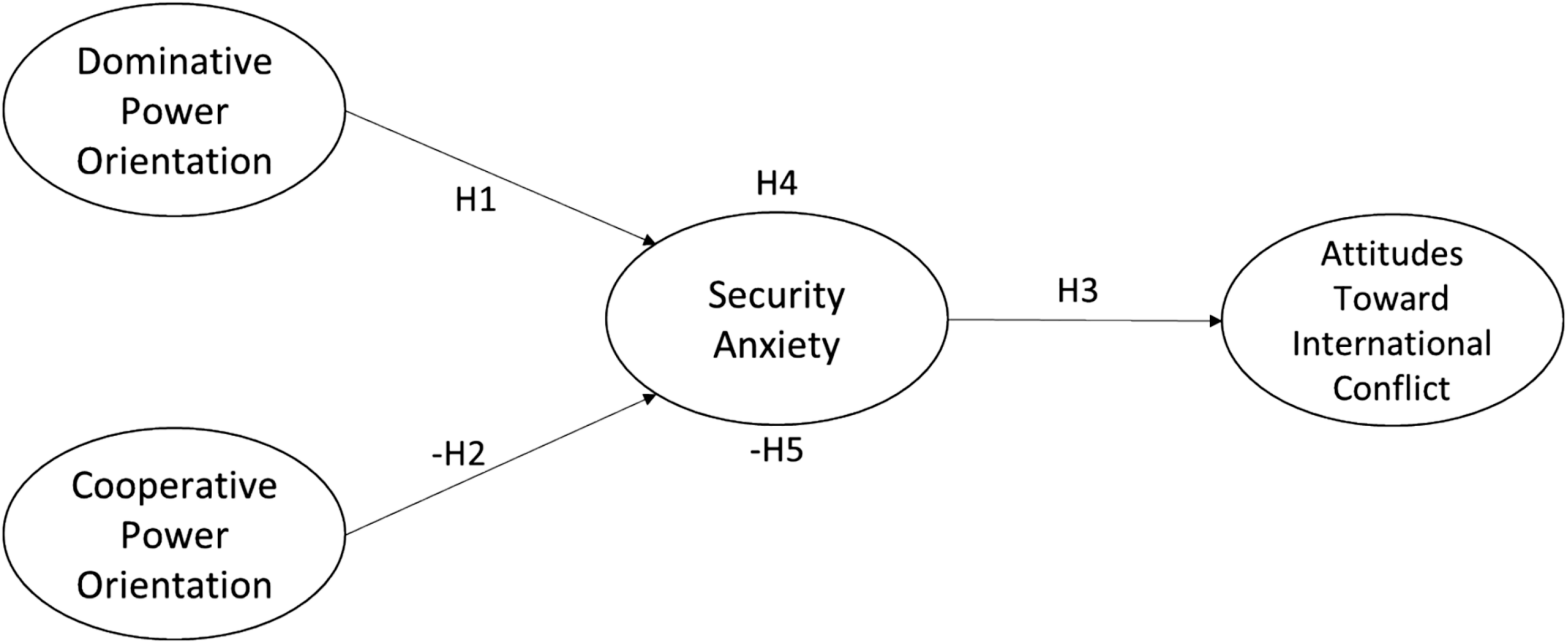
Research framework.

In summary, the conceptual framework of this study is illustrated in Figure 1, which integrates the relationships among power orientation, national security anxiety, and attitudes toward international conflict.

## 3 Research method

### 3.1 Research participants and data collection

This study targeted higher education students with foundational knowledge of international politics as the primary participants. Data were collected through a quantitative questionnaire survey to examine how dominative and cooperative power orientations influence national security anxiety and attitudes toward international conflict. A non-probability convenience sampling method was employed, focusing on undergraduate students in their third year or above, as well as graduate students majoring in international relations, political science, public affairs, diplomacy, or sociology. This group was deemed capable of understanding international issues and distinguishing political perspectives, thus suitable for responding to the abstract concepts involved in this study. The survey was administered online via the Wenjuanxing platform (similar to Qualtrics or SurveyMonkey). The questionnaire link was distributed through university teaching platforms, departmental groups, social media platforms, and email invitations. A total of 393 valid responses were collected. According to Hair et al. (2019), in Partial Least Squares Structural Equation Modeling (PLS-SEM), when the research model includes four latent variables and multiple structural paths, a sample size exceeding 200 is considered statistically adequate. Therefore, the sample obtained in this study meets the recommended threshold for statistical reliability.

While the use of convenience samples may raise concerns regarding external validity, prior research in political psychology has demonstrated that student samples remain appropriate for theory testing and examining underlying psychological mechanisms (Coppock and McClellan, 2019; Druckman and Kam, 2011). Moreover, empirical evidence suggests that findings derived from convenience samples are often comparable to those obtained from more diverse populations, particularly when the focus is on psychological relationships rather than population-level estimates (Mullinix et al., 2015). In this study, focusing on Chinese university students provides a meaningful and contextually rich perspective on the attitudes of Generation Z in a rapidly evolving geopolitical environment, although caution should be exercised when generalizing the findings to other populations or cultural contexts.

### 3.2 Research instruments

This study employed a structured questionnaire as the primary instrument for data collection. Based on the theoretical framework, the questionnaire comprised four major constructs and a set of demographic variables.

(1) Dominative Power Orientation (DPO)

The scale measuring Dominative Power Orientation was developed based on the theoretical perspectives of Mearsheimer (2001) and Tickner (1992) on the nature of power, emphasizing individuals’ endorsement of control, deterrence, and military superiority. The items were adapted from Duckitt et al. (2002) and their Hegemony Orientation Scale. Sample items include statements such as “A country must possess stronger military power than others to ensure its own security.” Respondents rated each item on a seven-point Likert scale ranging from 1 (“strongly disagree”) to 7 (“strongly agree”).

(2) Cooperative Power Orientation (CPO)

The Cooperative Power Orientation scale was designed according to Tickner’s (1992) feminist conception of power-sharing and cooperation, and adapted from Eagly and Wood’s (1991) Empowering Power Scale. This scale assessed respondents’ value orientation toward mutual trust, collaboration, and multilateral cooperation. Example items include “Stability in the international community should be built on cooperation and mutual trust” and “Disputes between nations should be resolved through dialogue rather than confrontation.”

(3) National security anxiety (NSA)

The national security anxiety scale measured individuals’ perceived anxiety and insecurity in response to international threats. Its theoretical basis was drawn from the research on political anxiety by Huddy et al. (2005) and Marcus et al. (2000). Representative items include “I worry that other countries may threaten our national sovereignty” and “Changes in the international situation make me feel uncertain about the future.” These items reflect respondents’ subjective perceptions of external threats and security concerns.

(4) Attitudes toward International Conflict (ICA)

The attitudes toward international Conflict scale was adapted from Mayton’s (2009) Peace and War Attitude Scale, which measures individuals’ preferences for military or diplomatic solutions in international disputes. It includes two opposing dimensions—militaristic (hawkish) and peace-oriented (dovish) attitudes. Example items include “I support the use of force to defend national interests against external threats” and “International conflicts should primarily be resolved through diplomacy and negotiation.” Items representing peace-oriented attitudes were reverse-coded during data analysis to maintain consistency in score direction.

Finally, the questionnaire collected participants’ demographic information, including gender, age, field of study, education level, whether they had taken courses related to international relations, political orientation, and frequency of exposure to international news. These variables were used to describe the sample characteristics and control for potential covariate effects in subsequent analyses.

Although the scales for Dominative Power Orientation and Cooperative Power Orientation were newly developed in this study, their construction was firmly grounded in established theoretical frameworks and prior empirical research. The items were adapted and refined based on existing constructs, such as Social Dominance Orientation and cooperative/empowering power perspectives. In addition, the measurement model demonstrated strong reliability and validity, suggesting that the scales are appropriate for capturing the intended constructs in this research context. In addition to statistical validation, the constructs in this study are conceptually grounded in established theoretical traditions. Dominative Power Orientation is rooted in realism and social dominance theory, while Cooperative Power Orientation draws on constructivist and feminist perspectives on relational power. National security anxiety is derived from political psychology literature on threat perception and anxiety, and Attitudes toward International Conflict reflect established distinctions between hawkish and dovish orientations. This theoretical grounding further supports the construct validity of the measures. The full questionnaire, including the original Mandarin version and its English translation, is provided in Appendix A in Supplementary material.

### 3.3 Data analysis methods

To test the hypothesized research model, this study employed Partial Least Squares Structural Equation Modeling (PLS-SEM) as the main analytical technique, using SmartPLS 4.0 for model estimation. PLS-SEM is particularly suitable for exploratory research and complex models involving multiple latent variables and mediating relationships. It also provides robust parameter estimates even with moderate sample sizes (Hair et al., 2021). Prior to analysis, the dataset was screened for missing values. Cases with incomplete responses on key measurement items were removed using listwise deletion to ensure the reliability of the constructs used in the SEM analysis. After data cleaning, a total of 393 valid responses were retained for subsequent analysis. The data analysis was conducted in three stages. First, preliminary analyses were performed using SPSS 26.0 for sample description and data preprocessing. This included examining demographic characteristics, calculating means and standard deviations of all variables, and testing for skewness and kurtosis to ensure normality. Outliers were also examined and treated where necessary. Second, the measurement model was evaluated to ensure reliability and validity. Item reliability was assessed using factor loadings (≥ 0.70), internal consistency was verified through composite reliability (CR ≥ 0.70), and convergent validity was confirmed via the average variance extracted (AVE ≥ 0.50). Discriminant validity was assessed using the Fornell–Larcker criterion, whereby the square root of the average variance extracted (AVE) for each construct should exceed its correlations with other constructs. These evaluation criteria were adopted to ensure the construct validity of the measurement model, including both convergent and discriminant validity, thereby supporting the robustness of the latent constructs used in this study. Third, the structural model was tested to examine the significance of hypothesized path coefficients, the explanatory power (*R*^2^ values) of endogenous constructs, and the mediating effect of national security anxiety (NSA). The bootstrapping procedure with 5,000 resamples was employed to test path significance and mediation effects. Additionally, variance inflation factor (VIF) values were checked to ensure the absence of multicollinearity among predictor variables.

## 4 Research results

### 4.1 Demographic profile of respondents

Table 1 presents the demographic characteristics of the participants in this study. A total of 393 valid questionnaires were collected. Among the respondents, 184 were male (46.8%) and 209 were female (53.2%), indicating a relatively balanced gender distribution. Regarding educational background, the majority were undergraduate students (44.0%), followed by master’s students (33.1%) and junior college students (11.7%), suggesting that the sample was primarily composed of individuals at higher education levels with sufficient academic literacy and understanding of international issues.

**Table 1:** Demographic characteristics of respondents.

Category	Group	Frequency	Percentage (%)
Gender	Male	184	46.8
	Female	209	53.2
Highest Education Level	Junior (third-year undergraduate)		
	Senior (fourth-year undergraduate)		
	Graduate and above (master’s or doctoral level)		
Have you taken courses related to international relations (including electives or compulsory)?	Yes	184	46.8
	No	209	53.2
Have you participated in academic activities or student organizations related to international politics, diplomacy, or global affairs?	Yes	145	36.9
	No	248	63.1
English Proficiency	No	53	13.5
	Basic conversational ability	125	31.8
	Intermediate (able to read general news)	108	27.5
	Fluent (able to communicate and read professional materials proficiently)	107	27.2
How often do you access international news on average?	Several times per day	117	29.8
	Once per day	77	19.6
	3–4 times per week	69	17.6
	1–2 times per week	83	21.1
	Less than once per month	47	12.0

In terms of international relations learning experience, 46.8% of the respondents had taken related courses, while 53.2% had not. Moreover, 36.9% reported participating in academic activities or student organizations related to international politics, diplomacy, or global affairs, indicating that a portion of the sample had practical exposure to international topics. As for English proficiency, the majority reported basic conversational ability (31.8%) or intermediate reading proficiency (27.5%), while 27.2% were able to use English fluently. This distribution suggests that most respondents possessed adequate language ability to comprehend international information. Concerning exposure to international news, nearly half of the respondents (49.4%) reported accessing international news several times per day or at least once daily, indicating a high level of engagement with global current affairs. About 12.0% of the participants reported following international news less than once per month, suggesting limited involvement among a small subgroup.

Overall, the sample consisted primarily of young adults with higher education backgrounds, moderate-to-high English proficiency, and strong interest in international issues. This demographic composition effectively reflects the characteristics of Chinese university students in the formation of their political attitudes and power orientations, thereby ensuring good representativeness for the study.

### 4.2 Convergent validity

According to the criteria proposed by Fornell and Larcker (1981), the measurement model should satisfy the following conditions to demonstrate convergent validity: (1) factor loadings should exceed 0.70, (2) composite reliability (CR) should be higher than 0.70, (3) the average variance extracted (AVE) should exceed 0.50, and (4) Cronbach’s *α* should be greater than 0.70. In this study, the factor loadings of all measurement items ranged from 0.854 to 0.948, exceeding the recommended threshold of 0.70. The composite reliability (CR) values ranged from 0.953 to 0.968, indicating excellent internal consistency across all constructs. The average variance extracted (AVE) values ranged from 0.803 to 0.859, surpassing the 0.50 criterion, while the Cronbach’s α coefficients ranged from 0.939 to 0.959, significantly above the 0.70 benchmark. Overall, these results confirm that the measurement model demonstrates strong convergent validity, as shown in Table 2.

**Table 2:** Convergent validity analysis.

Construct	Item	Factor loading	Cronbach’s alpha	Composite reliability (CR)	Average variance extracted (AVE)
Attitudes toward international conflict	ATIC1	0.804	0.859	0.892	0.580
	ATIC2	0.810			
	ATIC3	0.705			
	ATIC4	0.759			
	ATIC5	0.765			
	ATIC6	0.720			
Cooperative power orientation	CP1	0.816	0.817	0.879	0.644
	CP2	0.772			
	CP3	0.836			
	CP4	0.786			
Dominative power orientation	DP1	0.797	0.809	0.874	0.636
	DP2	0.793			
	DP3	0.853			
	DP4	0.742			
Security anxiety	SA1	0.869	0.860	0.903	0.700
	SA2	0.800			
	SA3	0.825			
	SA4	0.851			

### 4.3 Discriminant validity

This study assessed discriminant validity using the Average Variance Extracted (AVE) method. According to the criterion proposed by Fornell and Larcker (1981) discriminant validity is established when the square root of a construct’s AVE is greater than its correlations with other constructs. The analysis results indicate that, for most constructs, the square roots of the AVE values are significantly higher than the inter-construct correlation coefficients, satisfying the criterion for discriminant validity. As shown in Table 3, the measurement model in this study demonstrates adequate discriminant validity.

**Table 3:** Discriminant validity analysis (Fornell–Larcker criterion).

Construct	Attitudes toward international conflict	Cooperative power orientation	Dominative power orientation	Security anxiety
Attitudes toward international conflict	**0.761**			
Cooperative power orientation	−0.269	**0.803**		
Dominative power orientation	0.567	−0.369	**0.797**	
Security anxiety	0.521	−0.284	0.404	**0.837**

Bold values represent the square root of the average variance extracted (AVE) for each construct.

### 4.4 Goodness of fit

The Goodness of Fit (GOF) index is used to assess the overall quality of the measurement model and is calculated as: $\mathrm{GOF}=\sqrt{\bar{\mathrm{AVE}}}\;x\;\sqrt{\bar{R^{2}}}$ . According to Vinzi et al. (2010), GOF values of 0.10, 0.25, and 0.36 indicate small, medium, and large degrees of model fit, respectively. In this study, the calculated GOF value is 0.382, which exceeds the threshold for a strong fit. This result demonstrates that the research model exhibits a high level of overall goodness of fit and adequately explains the observed data.


$$\mathrm{GOF}=\sqrt{\bar{\mathrm{AVE}}}\;x\;\sqrt{\bar{R^{2}}}=\sqrt{0.640x\;0.228}=0.382$$

### 4.5 Path analysis

The results of the path analysis indicate that Security Anxiety is positively associated with Attitudes Toward International Conflict (*β* = −0.156, *t* = 2.779, *p* = 0.005), suggesting that individuals with a higher level of cooperative power orientation experience lower levels of national security anxiety. Conversely, Dominative Power Orientation shows a significant positive effect on Security Anxiety (*β* = 0.347, *t* = 6.105, *p* < 0.001), indicating that individuals with stronger dominative power orientations tend to exhibit greater security anxiety. Furthermore, Security Anxiety exerts a significant positive influence on Attitudes Toward International Conflict (*β* = 0.521, *t* = 11.949, *p* < 0.001), implying that heightened security anxiety amplifies individuals’ support for conflict-oriented or hawkish attitudes toward international disputes, as shown in Table 4.

**Table 4:** Path analysis results.

Path relationship	Path coefficient (*β*)	Standard deviation (SD)	*t*-value	*p*-value
Cooperative power orientation → security anxiety	−0.156	0.056	2.779	0.005
Dominative power orientation → security anxiety	0.347	0.057	6.105	0.000
Security anxiety → attitudes toward international conflict	0.521	0.044	11.949	0.000

**Figure 2:**
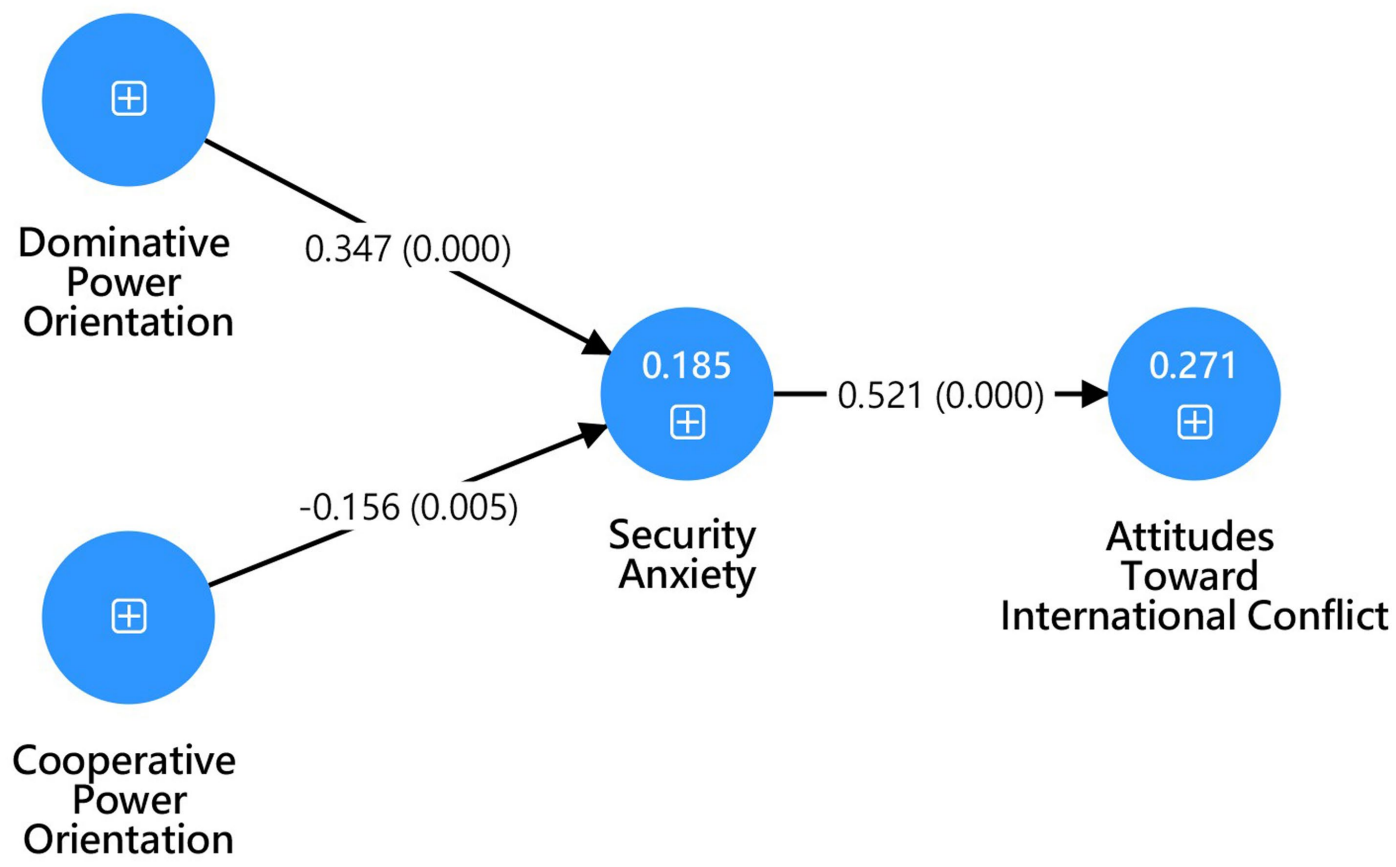
The proposed PLS-SEM model of power orientations, national security anxiety, and attitudes toward international conflict.

Figure 2 presents the empirical results of the Partial Least Squares Structural Equation Modeling (PLS-SEM) analysis.

### 4.6 Mediation effects

The results of the mediation analysis indicate that Cooperative Power Orientation (CPO) exerts a significant negative indirect effect on Attitudes Toward International Conflict (ATIC) through Security Anxiety (SA) (*β* = −0.082, SE = 0.028, *t* = 2.879, *p* = 0.004, 95% CI [−0.136, −0.025]). This finding suggests that individuals with a stronger cooperative orientation tend to experience lower levels of security anxiety, which in turn weakens their support for international conflict. Conversely, Dominative Power Orientation (DPO) shows a significant positive indirect effect on ATIC via Security Anxiety (*β* = 0.181, SE = 0.039, *t* = 4.619, *p* < 0.001, 95% CI [0.110, 0.265]). This result demonstrates that individuals with a higher dominative orientation tend to experience greater levels of security anxiety, which subsequently amplifies their favorable attitudes toward international conflict. Overall, these findings confirm the mediating role of security anxiety, highlighting its critical function as the emotional pathway through which cognitive power beliefs are translated into conflict-related attitudes, as shown in Table 5.

**Table 5:** Mediation analysis results.

Path relationship	Path coefficient (*β*)	Standard deviation	*t*-value	*p*- values	2.50%	97.50%
Cooperative power orientation → security anxiety → attitudes toward international conflict	−0.082	0.028	2.879	0.004	−0.136	−0.025
Dominative power orientation → ssssecurity Anxiety → attitudes toward international conflict	0.181	0.039	4.619	0.000	0.110	0.265

## 5 Conclusion and discussion

### 5.1 Research conclusion

#### 5.1.1 The effect of dominative power orientation on national security anxiety

The results indicate that Dominative Power Orientation (DPO) is positively associated with national security anxiety (NSA), supporting Hypothesis H1. This finding aligns with the central assumptions of realist theory, which posits that under conditions of anarchy, the international system is characterized by competition and threat, compelling states to rely on power superiority to ensure their security (Mearsheimer, 2019; Waltz, 1979). Individuals who internalize this “power-as-defense” mindset are more likely to interpret the external world through a threat-sensitive lens, leading to elevated levels of security anxiety. From a political psychology perspective, dominance-oriented beliefs amplify the “threat amplification effect”, wherein individuals exhibit heightened vigilance and defensive emotional responses when confronted with uncertainty or perceived danger (Feldman and Stenner, 1997; Huddy et al., 2005). This pattern suggests that those endorsing dominative power beliefs tend to equate control with safety, perceiving potential threats even in ambiguous contexts. Consequently, dominative power orientation serves as a psychological foundation for the activation of defensive emotions, linking structural realism’s security logic with individual-level affective responses.

The findings of this study also correspond with Duckitt’s (2001) Dual-Process Motivational Model, which posits that individuals high in Social Dominance Orientation (SDO) and Right-Wing Authoritarianism (RWA) tend to prefer hierarchical social structures and control over others, making them more sensitive to external threat cues. In psychological terms, dominative power orientation overlaps conceptually with a threat-oriented cognitive schema, predisposing individuals to perceive instability and vulnerability in national security contexts.

At the same time, prior research has demonstrated that psychological insecurity motivates individuals to seek authority and power as sources of protection, thereby reinforcing a reciprocal cycle of dominance and anxiety (Duckitt and Sibley, 2009). The present study empirically validates this theoretical linkage, suggesting that dominative power orientation may operate through an amplification of threat perception and expansion of uncertainty-related anxiety—forming a self-reinforcing psychological loop of “power–anxiety–defensiveness.”

Compared with existing studies, the innovative contribution of this research lies in empirically verifying the psychological foundations of traditional power theories at the individual level. While classical and offensive realism primarily emphasize power competition and security dilemmas at the state level (Morgenthau et al., 1985; Schweller, 2016), this study reveals that dominative power orientation, as an internalized cognitive tendency, can trigger similar mechanisms of security anxiety at the micro level. This finding not only illustrates how power beliefs permeate individual emotional structures but also helps to bridge the theoretical gap between international relations theory and political psychology.

Notably, the significant impact of dominative power orientation on security anxiety among Chinese university students can also be explained by socio-cultural factors. China’s younger generation has grown up in a period marked by intensified globalization and geopolitical tension, where external threat narratives—such as Sino–U.S. rivalry and sovereignty disputes—are widely present in media discourse and educational contexts. Consequently, many young individuals have subconsciously internalized the cognitive schema that “security must be maintained through strength.” This process of political socialization makes it easier for dominative power orientation to transform into emotional insecurity, resulting in heightened levels of national security anxiety.

#### 5.1.2 The effect of cooperative power orientation on national security anxiety

The results indicate that cooperative power orientation exerts a significant negative effect on national security anxiety, supporting Hypothesis H2. This finding suggests that individuals who hold a cooperative power orientation tend to interpret international relations through a framework centered on mutual trust, norms, and communication, thereby perceiving lower levels of external threat and experiencing reduced anxiety.

This result aligns with the perspectives of constructivist and feminist international relations theories, both of which emphasize that interactions within the international system are shaped not solely by material capabilities but also by shared ideas, norms, and identities (Tickner, 1992; Wendt, 1992). When individuals conceptualize power as the capacity to cooperate rather than the means to dominate, their sense of security derives from mutual dependence and institutional trust rather than from military superiority or deterrence. Consequently, cooperative power beliefs mitigate the emotional responses associated with uncertainty and threat, effectively lowering national security anxiety (Adler and Pouliot, 2011).

Theoretically, the cooperative power orientation reflects a *relational security logic*. In contrast to the zero-sum thinking characteristic of the dominative power orientation, the cooperative orientation emphasizes the possibility of *positive-sum relationships*, where national security can be achieved through multilateral cooperation, communication, and normative consensus. Barnett and Duvall (2005) argue that power does not exist solely within coercive structures but also manifests in the interactive practices of norms, discourse, and identity. Therefore, when individuals perceive power as a *process of co-governance (power with)* rather than a *means of control (power over)*, their emotional responses to the international environment tend to be more stable. Eagly and Wood (1991) also found that individuals with a cooperative orientation display higher levels of trust and *integrative thinking* in social interactions. When facing uncertainty, such individuals respond to potential threats in a more open and rational manner, thereby reducing anxiety levels.

The findings of this study are also consistent with previous research in political psychology. Halperin (2011) suggested that individuals with higher levels of empathy and cooperative beliefs tend to activate an *emotional buffer mechanism* when encountering conflict-related information. Through positive interpretations of others’ intentions and rational evaluations of situational threats, these individuals can reduce the intensity of fear and anxiety. The present study’s results support this view, indicating that cooperative power orientation functions as a belief system for emotional regulation, which mitigates defensive reactions and enhances a sense of psychological security when facing external threats.

Compared with prior research that has primarily focused on the macrosocial and institutional effects of cooperative power on political outcomes (Keohane, 1984; Nye, 2011). This study makes an innovative contribution by empirically verifying its *emotional buffering function* at the individual psychological level. The empirical evidence demonstrates that cooperative power orientation is not merely a normative ideal but a concrete psychological mechanism capable of reducing national security anxiety. This finding extends the theoretical boundaries of constructivist perspectives on “trust generation,” highlighting how power beliefs shape subjective experiences of international security through the interaction of cognitive frameworks and emotional responses.

Regarding the study sample, the findings from Chinese university students are particularly revealing. Compared with individuals with a stronger dominative orientation, those with higher cooperative power orientation tend to interpret international order through institutional trust and multilateral cooperation, developing greater empathy and cross-cultural understanding through globalized education and intercultural experiences. This suggests that the younger generation, influenced by an increasingly open educational and informational environment, is gradually internalizing values that favor dialogue over confrontation and cooperation over conflict. Such a pattern not only reflects a shifting mindset among contemporary Chinese youth toward global governance issues but also illustrates how cooperative power orientation, as a psychological resource, fosters emotional stability and rational judgment in contexts of perceived threat.

#### 5.1.3 The effect of national security anxiety on attitudes toward international conflict

The results indicate that national security anxiety has a significant positive effect on attitudes toward international conflict, supporting Hypothesis H3. This finding suggests that when individuals experience heightened perceptions of security threats and uncertainty, they are more likely to support assertive or military-oriented policies as a means of restoring both psychological and structural security.

This result is consistent with the Affective Intelligence Theory in political psychology, which posits that anxiety activates an individual’s surveillance system, increasing attention to risks and motivating protective or defensive behaviors (Marcus et al., 2000). As security anxiety rises, emotion-driven threat appraisals tend to replace rational analysis, leading to greater support for strong leadership and coercive policies (Huddy et al., 2005). In essence, national security anxiety functions as a mobilizing emotion that channels fear and uncertainty into preference for aggressive responses. This emotional mechanism underscores how perceived insecurity transforms into hawkish attitudes toward conflict, reflecting the broader psychological dynamics through which emotions shape political decision-making and foreign policy orientation.

Compared with existing literature, the findings of this study are consistent with Brader et al. (2008), who demonstrated that anxiety significantly strengthens distrust toward out-groups and increases support for military mobilization, while simultaneously weakening confidence in diplomatic compromise. Similarly, Gross et al. (2017), in their research on cyberterrorism, found that highly anxious individuals are more likely to endorse restrictive and retaliatory policies. Together, these studies underscore the powerful mobilizing function of anxiety in shaping political attitudes. The present study extends this understanding to the domain of national security, confirming that anxiety operates in a similar psychological manner within international conflict contexts—transforming perceptions of insecurity into heightened support for coercive and militarized responses.

From a theoretical perspective, the influence of anxiety on conflict attitudes can be explained through two interrelated pathways. The first is the threat-response mechanism, in which anxiety motivates individuals to adopt defensive and deterrent strategies as a means of reducing uncertainty and restoring a sense of control (Albertson and Gadarian, 2015). The second is the emotional rationalization pathway, whereby anxiety leads individuals to reinforce stereotypical perceptions of hostile out-groups, thereby rationalizing the necessity of military actions (Marcus et al., 2000). In other words, anxiety not only shapes behavioral tendencies but also reconfigures belief systems, prompting individuals to psychologically accept policy frameworks such as “stability through strength” or “peace through deterrence.” This dual-path explanation underscores how emotions serve both as motivational forces and cognitive justifications in the formation of attitudes toward international conflict.

The findings of this study also reveal significant socialization differences within the sample. For Chinese university students, issues of national security have become highly salient in recent years due to the strong emphasis placed by state media and the education system on security narratives and “external threat framing.” Within this sociopolitical context, anxiety is more likely to merge with patriotic emotions and collective identity, giving rise to a psychological tendency toward defensive nationalism (Gries, 2004). Consequently, when anxiety is activated, its effects go beyond an emotional response to perceived threats—it becomes a policy preference shaped by collective consciousness. This study confirms that, under such a social context, national security anxiety significantly promotes hawkish attitudes toward international conflict.

Overall, this research highlights the central role of anxiety in shaping attitudes toward international conflict, empirically validating a “emotion–policy preference” psychological transmission model. Unlike previous studies that examined war support primarily from a macro-political or national sentiment perspective, this study, from a micro-level psychological standpoint, demonstrates that anxiety is not merely a fear reaction but a mediating construct that transforms cognitive and emotional interactions into concrete policy orientations. For Chinese youth, these findings suggest that security-related emotions have become a key psychological lens for understanding their international attitudes, while also implying that balancing security awareness with rational deliberation should be a critical goal for future international education and media communication strategies.

#### 5.1.4 The mediating role of national security anxiety

The findings demonstrate that national security anxiety serves as a significant mediating variable between power orientations and attitudes toward international conflict, supporting Hypotheses H4 and H5. In other words, a dominative power orientation increases security anxiety, which in turn reinforces hawkish or militarized attitudes, whereas a cooperative power orientation alleviates anxiety, thereby reducing support for military action. This result provides empirical support for the proposed “cognition–emotion–behavior” framework, illustrating that power beliefs influence not only individuals’ international attitudes but also their concrete policy preferences through emotional mechanisms.

From a theoretical standpoint, these results resonate with the affective mediation model in political psychology, which posits that emotions play a transmission role between cognitive beliefs and political behavior (Brader et al., 2008; Marcus et al., 2000). Individuals with a dominative power orientation possess a threat-oriented cognitive structure that heightens sensitivity to external risks, triggering anxiety responses and, consequently, stronger support for aggressive or defensive policies. Conversely, those with a cooperative power orientation, who interpret international interactions through trust and dialogue, are more capable of regulating anxiety and thus favor diplomatic and peaceful resolutions. These findings align with Halperin (2011) and Albertson and Gadarian (2015), who argue that anxiety is not merely an affective state but a psychological mechanism with explanatory power in shaping political attitudes. By highlighting the mediating role of security anxiety, this study bridges cognitive and emotional dimensions in understanding how underlying power beliefs translate into international conflict attitudes, offering a more integrated explanation of political emotion and behavior.

Furthermore, the mediating effects identified in this study lend additional support to the constructivist interpretation of power socialization. As Adler-Nissen and Pouliot (2014) argue, power is not merely a form of material control but a social relationship continuously reproduced through interactive practices. The findings of this study demonstrate that power beliefs become internalized within individuals’ psychological structures through emotional representation, resulting in distinct perceptions of security and behavioral tendencies across different power orientations. This provides empirical evidence that “emotionalized power” serves as a crucial mechanism for understanding individual differences in attitudes toward international conflict.

Compared with prior research, the innovation of this study lies in conceptualizing national security anxiety as a mediating mechanism of psychological security dilemma. Traditional international relations studies have primarily focused on state-level security dilemmas, such as the structure of mutual distrust emphasized by Jervis (1978). In contrast, this study translates the logic of the security dilemma to the individual level, revealing how security anxiety psychologically reproduces the logic of structural insecurity within the human mind. This finding bridges the theoretical gap between classical realism and political psychology, illustrating how cognitive beliefs and emotional processes jointly construct international behavioral attitudes.

Regarding the study sample, the mediating effects observed among Chinese university students carry notable theoretical implications. As international conditions evolve and narratives of external threat become increasingly prevalent, the power beliefs and security-related emotions of young individuals are more susceptible to influence through socialization processes. The findings show that students with a high dominative power orientation respond more intensely to perceived external threats, with anxiety serving as a key emotional driver behind their hawkish policy preferences. In contrast, those with a high cooperative power orientation tend to place greater trust in multilateral institutions and international cooperation, enabling them to evaluate international conflicts with greater emotional stability. This indicates that anxiety functions as a psychologically differentiating mechanism between the two power belief systems.

Overall, the study demonstrates that national security anxiety plays a central mediating role linking power orientation and conflict attitudes, revealing the internal psychological process through which power beliefs are transformed into policy stances. These findings deepen our understanding of the psychological effects of power orientation and contribute a micro-level extension to international relations theory, highlighting that the interaction among emotion, power, and attitude constitutes a critical and often overlooked pathway in contemporary political psychology research.

### 5.2 Discussion

#### 5.2.1 Academic contributions

The main academic contribution of this study lies in integrating international relations theory with political psychology, revealing from a micro-level perspective how power orientations influence individuals’ attitudes toward international conflict through emotional processes. The study proposes a novel “Power Orientation–National Security Anxiety–Conflict Attitude” psychological transmission model, expanding the explanatory capacity of both realist and constructivist theories while enriching emotion theory within the context of international political behavior.

First, on a theoretical level, this study fills a major gap in international relations theory by addressing the individual-level dynamics of power belief internalization. Traditional realism and offensive realism primarily focus on the competition for power among states, neglecting how individuals internalize and psychologically reproduce power beliefs. By operationalizing dominative and cooperative power orientations as measurable psychological constructs, this study demonstrates that these orientations, respectively, influence national security anxiety through threat perception and trust generation, which in turn shape conflict attitudes.

This finding illustrates that power is not only an external structural variable of the international system but also a cognitive belief system capable of influencing emotion and decision-making at the individual level. It thereby extends the theoretical applications of power frameworks proposed by Mearsheimer (2001) and Tickner (1992) beyond state behavior, introducing a new dimension that connects cognitive structures with emotional mechanisms in explaining political behavior.

Secondly, this study deepens the cross-theoretical application of the affective mediation model in political psychology. Previous emotion-based theories have primarily focused on domestic political behavior, such as voting decisions or policy support (Huddy et al., 2005; Marcus et al., 2000), with relatively little extension to issues of international security. This study confirms that national security anxiety plays a key mediating role between power orientation and conflict attitudes, revealing that emotion is not merely a secondary reaction to political events but a central transmission mechanism linking beliefs and attitudes. This finding expands the theoretical horizon of affective politics, offering a new analytical perspective for understanding the psychological mechanisms underlying international behavior. It highlights that emotional processes—particularly anxiety—serve as critical bridges between cognition and behavior in foreign policy attitudes, thereby enriching both international relations theory and political psychology with a shared interdisciplinary framework.

Third, this study proposes a dual-path psychological model of power orientation, juxtaposing the two mechanisms of “Dominative Power Orientation → Anxiety → Hawkish Attitude” and “Cooperative Power Orientation → Trust → Dovish Attitude.” This bidirectional framework establishes a symmetrical model of how power beliefs shape policy preferences, enabling a more balanced interpretation of individual behavioral differences under conditions of perceived security threat. The model not only explains why individuals exhibit divergent responses—ranging from aggressive to conciliatory tendencies—but also provides a quantitative foundation for future research on cooperative power psychology, addressing the longstanding theoretical bias toward domination and deterrence logics in international relations.

Fourth, this study offers empirical support for constructivist and feminist international relations theories. Through the operationalization and validation of cooperative power orientation, the findings demonstrate that the theoretical propositions of “power as social practice” and “power as empowerment” (Adler and Pouliot, 2011; Tickner, 1992) manifest concretely at the individual level of emotional experience and policy preference. This suggests that normative power and relational security logic are not confined to the diplomatic or institutional domains but can be internalized and reproduced as everyday psychological mechanisms, bridging the gap between macro-level theories of global governance and micro-level emotional cognition.

Finally, in terms of research subjects, this study employs Chinese university students as its sample, which carries significant academic and contemporary relevance. The youth generation represents a critical stage of political socialization, and the formation of their power orientations and security perceptions constitutes the psychological foundation for a nation’s future foreign policy mindset. The results reveal that under the dual contexts of globalization and geopolitical competition, Chinese youth exhibit both a heightened sensitivity to external threats and a latent inclination toward cooperation and institutional trust. This coexistence of a dominative security mindset and a cooperative global outlook reflects an evolving trajectory in how the younger generation conceptualizes power, providing a valuable theoretical reference for future studies on youth political psychology and national security perception.

In summary, the academic contribution of this study lies in the establishment of an integrative theoretical framework that connects power orientation, emotional processes, and international attitudes. By providing empirical evidence on how power beliefs are associated with conflict stances through national security anxiety, this research enriches the behavioral foundations of international relations theory from a psychological perspective. This cross-theoretical integration not only deepens the understanding of power psychology but also opens new pathways for exploring the emerging field of emotionalized international relations, where cognition, emotion, and power dynamics jointly shape global political behavior.

#### 5.2.2 Practical implications

The practical contributions of this study are reflected in three main areas: national security education, youth international mindset development, and optimization of public communication strategies. By uncovering the psychological mechanisms linking power orientation and security anxiety, this study provides policymakers and educational institutions with concrete insights into how to cultivate a more inclusive and rational sense of security across different social groups characterized by distinct power beliefs.

First, in the realm of national security education, the study reveals that individuals with a dominative power orientation are more sensitive to threats and prone to anxiety, making them more likely to support coercive or militarized policies when faced with uncertainty. This finding suggests that governments and educational institutions should avoid fear-based “threat-driven mobilization” in security education and instead adopt a constructive security awareness strategy. Such an approach emphasizes the importance of dialogue, institutional trust, and international cooperation, alongside sovereignty and defense education. By doing so, it prevents security issues from being oversimplified into emotional dichotomies of “us versus them” and fosters a mature, rational understanding of national security.

Second, regarding the cultivation of youth international perspectives, this study—based on a sample of Chinese university students—illustrates how the younger generation’s power psychology is structured within a globalized political environment. The findings show that students with a cooperative power orientation tend to interpret international conflicts with greater openness and balance, while experiencing lower levels of anxiety. This has strong implications for higher education and youth diplomacy training. Universities and policy institutes can integrate concepts of cooperative power and multilateralism into courses on international relations, diplomatic negotiation, and intercultural communication. Such educational designs would strengthen students’ trust in international institutions and peace mechanisms, fostering a rational, confident, and non-confrontational global citizenship mindset.

Third, at the level of public communication and media strategy, the study highlights the emotional transmission effects embedded in national security discourse, showing that the narrative framing of media messages directly shapes collective anxiety and public policy preferences. This has dual implications for both government and media organizations. On one hand, policy communication should balance risk communication with emotional reassurance, avoiding the overemphasis of conflict and threat narratives that may exacerbate public anxiety. On the other hand, public media discourse should amplify symbols and narratives that reinforce a cooperative power orientation, such as “shared security,” “a community with a shared future for humankind,” and “multilateral trust-building.” Through emotionally positive framing, these strategies can enhance societal resilience and promote public support for peaceful approaches to conflict resolution.

Finally, from a cross-theoretical application perspective, this study contributes a new paradigm of psychological policy analysis for decision-makers. By understanding how different power orientations shape anxiety and policy attitudes, policymakers can more effectively tailor message framing to reduce collective insecurity and promote rational policy responses. This analytical framework is not only applicable to national security and foreign policy, but can also be extended to domains such as public crisis management, environmental security, and technology governance, providing a psychologically grounded reference model for intersectoral policy design.

In summary, the practical significance of this study lies in its development of an applied framework centered on the “Power Psychology–Security Anxiety–Attitudinal Response” nexus. This framework helps policymakers and educators maintain a balance between security consciousness and cooperative values when addressing uncertainty and risk, promoting a more rational and emotionally balanced approach to national security education and public communication.

### 5.3 Research limitations and future directions

Despite its theoretical and empirical innovations, this study has several limitations that warrant consideration.

First, regarding the sample scope, this research focused on Chinese university students as the primary respondents. While this group effectively represents a core segment of youth political socialization, its cultural and geographical homogeneity limits the generalizability of the findings and constrains the external validity of the study. In particular, the results should be interpreted within the specific sociopolitical and cultural context of China and may not fully reflect the perceptions of young generations in other countries, especially in Western contexts. From this perspective, the findings of this study are best understood as context-specific. Future research could employ cross-cultural comparisons to examine whether the relationships identified in this study remain consistent across different national and cultural settings.

Second, in terms of research methodology, this study adopted a cross-sectional survey design, which captures only static associations between variables and cannot reveal causal dynamics. Future studies could utilize longitudinal or experimental designs to explore how external events, media exposure, or geopolitical developments dynamically affect anxiety levels and conflict attitudes over time.

Third, concerning variable operationalization, the scales for Dominative Power Orientation and Cooperative Power Orientation were newly developed in this study. Although they demonstrated satisfactory reliability and validity, they remain in an initial validation phase. Subsequent research could incorporate qualitative interviews, implicit association tests (IATs), or experimental priming techniques to more comprehensively capture individuals’ underlying power beliefs and emotional responses.

Finally, in terms of theoretical and practical extensions, future research may integrate frameworks such as Social Identity Theory or Social Dominance Theory to deepen understanding of the social-psychological foundations of power orientation. Additionally, applying this model to specific policy contexts—such as diplomatic crises, global technological competition, or environmental security—could help verify the mobilizing effects of national security anxiety in various issue domains.

In sum, future research can advance the field by employing cross-cultural sampling, multi-method approaches, and dynamic analyses to further validate the “Power Orientation–Emotion–Attitude” transmission mechanism, thereby enriching the psychological foundations of international behavioral research.

## Data Availability

The datasets generated and analyzed in this study are openly available in the Zenodo repository at https://doi.org/10.5281/zenodo.17597358.
