# Supplementary material for: Power, anxiety, and conflict: how dominative and cooperative orientations shape attitudes toward international relations
Source: Front Psychol. 2026 Apr 20;17:1770594. doi: 10.3389/fpsyg.2026.1770594 (PMC13139938; doi:10.3389/fpsyg.2026.1770594)
Supplement: Supplementary file 1 [file Supplementary_file_1.pdf]

Appendix A. Survey Instrument

All items were originally developed in Mandarin and translated into English for reporting purposes.

A1. Introduction（中英文对照）

中文：
亲爱的参与者您好：
您好！本问卷旨在了解大学生对「国际政治中权力观与冲突态度」的看法。问卷数据仅供学术研究使用，完全匿名，不涉及个人隐私信息，请您放心作答。问卷约需 8–10 分钟完成，所有题项皆无对错，请依据您真实的感受填写。
非常感谢您的协助！

English:
Dear Participant,
This questionnaire aims to investigate university students' perceptions of power orientations and attitudes toward international conflict. All responses are anonymous and will be used solely for academic research purposes. The survey takes approximately 8–10 minutes to complete. There are no right or wrong answers—please respond based on your true feelings.
Thank you for your participation.

A2. Demographic Information

中文	English
性别	Gender
☐ 男 ☐ 女 ☐ 其他 ☐ 不愿透露	☐ Male ☐ Female ☐ Other ☐ Prefer not to say
年龄：____岁	Age: ____ years
就读专业	Major
☐ 国际关系 ☐ 政治学 ☐ 社会学 ☐ 历史 ☐ 商学/经济 ☐ 其他	☐ International Relations ☐ Political Science ☐ Sociology ☐ History ☐ Business/Economics ☐ Other
学历层次	Education Level
☐ 大学本科 ☐ 硕士研究生 ☐ 博士研究生	☐ Undergraduate ☐ Master's ☐ Doctoral
是否修读国际关系课程	Have you taken IR-related courses?

☐ 是 ☐ 否	☐ Yes ☐ No
政治立场	Political orientation
☐ 保守 ☐ 中间 ☐ 自由 ☐ 不确定	☐ Conservative ☐ Moderate ☐ Liberal ☐ Uncertain
国际新闻接触频率	Frequency of following international news
☐ 每天 ☐ 每周数次 ☐ 偶尔 ☐ 几乎不看	☐ Daily ☐ Several times a week ☐ Occasionally ☐ Rarely

A3. Measurement Scale

Scale:1 = Strongly Disagree → 7 = Strongly Agree

A. Dominative Power Orientation

Item	中文	English
A1	国家必须拥有比其他国家更强的军事力量，才能保障自身安全。	A country must possess stronger military power than others to ensure its security.
A2	在国际政治中，只有强者才有发言权。	In international politics, only powerful states have a voice.
A3	如果他国威胁我方利益，我们应该立即展现军事实力。	If another country threatens our interests, we should immediately demonstrate military strength.
A4	权力就是能够控制他国行为的能力。	Power is the ability to control the behavior of other countries.

B. Cooperative Power Orientation

Item	中文	English
B1	国际社会的稳定应建立在合作与互信的基础上。	International stability should be based on cooperation and mutual trust.
B2	权力的本质是促进人与人之间的协作与共荣。	The essence of power lies in promoting cooperation and mutual prosperity.
B3	国与国之间应该以对话而非对抗解决分歧。	Countries should resolve disagreements through dialogue rather than confrontation.
B4	我相信通过多边合作能提升全球安全。	I believe multilateral cooperation enhances global security.

C. National Security Anxiety

Item	中文	English
C1	我担心其他国家会对我们国家的主权造成威胁。	I worry that other countries may threaten our national sovereignty.
C2	国际局势的变化让我对未来感到不安。	Changes in the international situation make me feel anxious about the future.
C3	我认为我们国家随时有可能面临军事冲突。	I believe our country may face military conflict at any time.
C4	国防准备不足会让我对国家安全感到焦虑。	Insufficient defense preparedness makes me feel anxious about national security.

D. Attitudes Toward International Conflict

Item	中文	English
D1	为了国家利益，我支持动用武力对抗威胁。	I support the use of force to counter threats for national interests.
D2	某些时候，战争是不可避免的正当选择。	In some cases, war is an inevitable and justified choice.
D3	军事实力是解决国际纠纷的最佳方式。	Military power is the best way to resolve international disputes.
D4	国际争端应优先通过外交与协商解决。（反向）	International disputes should be resolved through diplomacy and negotiation first. (reverse)
D5	政府应积极参与多边和平机制以防止冲突。（反向）	Governments should actively participate in multilateral peace mechanisms. (reverse)
D6	国与国应寻求共识而非竞争以维护和平。（反向）	Countries should seek consensus rather than competition to maintain peace. (reverse)
